# Transposase-assisted tagmentation: an economical and scalable strategy for single-worm whole-genome sequencing

**DOI:** 10.1093/g3journal/jkae094

**Published:** 2024-06-10

**Authors:** Zi Wang, Jingyi Ke, Zhengyang Guo, Yang Wang, Kexin Lei, Shimin Wang, Guanghan Chen, Zijie Shen, Wei Li, Guangshuo Ou

**Affiliations:** State Key Laboratory of Membrane Biology, Tsinghua-Peking Center for Life Sciences, Beijing Frontier Research Center for Biological Structure, School of Life Sciences and MOE Key Laboratory for Protein Science, McGovern Institute for Brain Research, Tsinghua University, Beijing 100190, China; State Key Laboratory of Membrane Biology, Tsinghua-Peking Center for Life Sciences, Beijing Frontier Research Center for Biological Structure, School of Life Sciences and MOE Key Laboratory for Protein Science, McGovern Institute for Brain Research, Tsinghua University, Beijing 100190, China; State Key Laboratory of Membrane Biology, Tsinghua-Peking Center for Life Sciences, Beijing Frontier Research Center for Biological Structure, School of Life Sciences and MOE Key Laboratory for Protein Science, McGovern Institute for Brain Research, Tsinghua University, Beijing 100190, China; State Key Laboratory of Membrane Biology, Tsinghua-Peking Center for Life Sciences, Beijing Frontier Research Center for Biological Structure, School of Life Sciences and MOE Key Laboratory for Protein Science, McGovern Institute for Brain Research, Tsinghua University, Beijing 100190, China; State Key Laboratory of Membrane Biology, Tsinghua-Peking Center for Life Sciences, Beijing Frontier Research Center for Biological Structure, School of Life Sciences and MOE Key Laboratory for Protein Science, McGovern Institute for Brain Research, Tsinghua University, Beijing 100190, China; State Key Laboratory of Membrane Biology, Tsinghua-Peking Center for Life Sciences, Beijing Frontier Research Center for Biological Structure, School of Life Sciences and MOE Key Laboratory for Protein Science, McGovern Institute for Brain Research, Tsinghua University, Beijing 100190, China; State Key Laboratory of Membrane Biology, Tsinghua-Peking Center for Life Sciences, Beijing Frontier Research Center for Biological Structure, School of Life Sciences and MOE Key Laboratory for Protein Science, McGovern Institute for Brain Research, Tsinghua University, Beijing 100190, China; State Key Laboratory of Membrane Biology, Tsinghua-Peking Center for Life Sciences, Beijing Frontier Research Center for Biological Structure, School of Life Sciences and MOE Key Laboratory for Protein Science, McGovern Institute for Brain Research, Tsinghua University, Beijing 100190, China; School of Medicine, Tsinghua University, Beijing 100190, China; State Key Laboratory of Membrane Biology, Tsinghua-Peking Center for Life Sciences, Beijing Frontier Research Center for Biological Structure, School of Life Sciences and MOE Key Laboratory for Protein Science, McGovern Institute for Brain Research, Tsinghua University, Beijing 100190, China

**Keywords:** whole-genome sequencing, transposase-assisted tagmentation, single-worm

## Abstract

AlphaMissense identifies 23 million human missense variants as likely pathogenic, but only 0.1% have been clinically classified. To experimentally validate these predictions, chemical mutagenesis presents a rapid, cost-effective method to produce billions of mutations in model organisms. However, the prohibitive costs and limitations in the throughput of whole-genome sequencing (WGS) technologies, crucial for variant identification, constrain its widespread application. Here, we introduce a Tn5 transposase-assisted tagmentation technique for conducting WGS in *Caenorhabditis elegans*, *Escherichia coli*, *Saccharomyces cerevisiae*, and *Chlamydomonas reinhardtii*. This method, demands merely 20 min of hands-on time for a single-worm or single-cell clones and incurs a cost below 10 US dollars. It effectively pinpoints causal mutations in mutants defective in cilia or neurotransmitter secretion and in mutants synthetically sterile with a variant analogous to the B-Raf Proto-oncogene, Serine/Threonine Kinase (BRAF) V600E mutation. Integrated with chemical mutagenesis, our approach can generate and identify missense variants economically and efficiently, facilitating experimental investigations of missense variants in diverse species.

## Teaser

Wang et al. introduce Transposase-Assisted Tagmentation, a cost-effective method for whole-genome sequencing (WGS) in model organisms. This method, utilizing Tn5 transposase, requires minimal hands-on time and low cost. It effectively identifies causal mutations in mutants and is applicable to various species like yeast and algae. Overall, the authors’ method offers an economical and efficient approach for generating and identifying missense variants in diverse species.

## Introduction

Utilizing Artificial Intelligence, AlphaFold2 has formulated billions of protein structure models, elucidating protein structure–function relationships crucial to understanding organismal biology, while simultaneously advancing our knowledge of diseases and facilitating the development of novel therapeutics (Jumper *et al.* 2021). Recent advancements with AlphaMissense, a deep learning model built upon AlphaFold2, have categorized 89% of an estimated 71 million possible missense variants within the human proteome as either likely pathogenic or benign (Cheng *et al.* 2023). However, a mere 0.1% of such predictions have been corroborated by clinical data or functional studies (Cheng *et al.* 2023). Though clustered regularly interspaced short palindromic repeats (CRISPR)–Cas9-based genome editing strategies have been widely adopted to create genome-edited cell lines and animals to explore the implications of human missense mutations in model organisms (Cong *et al.* 2013; Mali *et al.* 2013; Adli 2018; Bock *et al.* 2022), the generation of single-amino acid substitutions across the cell or animal models usually requires months of experimentation and involves costs upwards of several thousand US dollars, operating in a one-at-a-time manner. This approach contrasts starkly with the original aim of high-throughput loss-of-function or gain-of-function genetic screens that target the genome as a whole. Thus, there emerges a critical need to establish a rapid, economical, and scalable methodology to experimentally explore the physiological or pathological relevance of human missense variants.

Chemical mutagenesis, utilizing agents such as the alkylating compound Ethyl Methane Sulfonate (EMS) to alter DNA sequences and induce mutations, has been broadly employed across species in genetic and genomic research for decades (Sega 1984; Jorgensen and Mango 2002; Page and Grossniklaus 2002). For instance, Sydney Brenner pioneered the use of chemical mutagenesis in the model organism *Caenorhabditis elegans* (*C. elegans*) in the 1970s (Brenner 1974). Adhering to the Brenner's protocol, a single round of chemical mutagenesis typically yields ∼91 missense variants among roughly 419 genomic alterations in an individual worm (Thompson *et al.* 2013). The simplicity of culturing nematodes facilitates the chemical mutagenesis of 1 million individual worms within just 1 week (Jorgensen and Mango 2002), all at a nearly negligible cost. Since each mutagenized *C. elegans* produces about 100 progenies, chemical mutagenesis can thereby generate billions of missense variants both rapidly and economically (Liu *et al.* 1999). The same holds when chemical mutagenesis is applied to other model organisms, including bacteria (Cupples and Miller 1989; Cupples *et al.* 1990), yeast (Winston 2008), and algae (Loppes 1969). Despite the potential and historical use of chemical mutagenesis, a major bottleneck impeding its widespread application is the financial burden associated with whole-genome sequencing (WGS) platforms, which are vital for identifying variants. A testament to this challenge is the Million Mutation Project, undertaken by the collective efforts of the community 12 years ago (Thompson *et al.* 2013). This project, which sequenced 2,007 chemically or UV mutagenized *C. elegans*, identified ∼183,327 missense variants at a considerable expense of several million US dollars (Thompson *et al.* 2013). Despite its significance, the project was not continued, in part due to the then-prohibitive cost of WGS per strain, amounting to about 10,000 US dollars. Although the cost of WGS for a *C. elegans* strain has since diminished to several hundred US dollars, sequencing millions of strains remains economically unfeasible.

Beyond cost reduction, equally crucial for the application of chemical mutagenesis is minimizing the biological samples required to generate a WGS library. In a typical forward genetic screen of homozygous F2 *C. elegans*, only ∼10% generate live F3 progenies with heritable phenotypes (Jorgensen and Mango 2002). Even when exhibiting notable phenotypes, the remaining F2 mutant animals might, regrettably, be sterile or lethal that does not transmit to subsequent generations (Jorgensen and Mango 2002). While theoretically plausible, maintaining heterozygotic mutants using a genetic balancer proves to be operationally tedious and is not universally applicable to every mutant. Thus, there is a significant demand to collect individual mutant worms to glean their genome information through WGS sequencing.

Bacterial transposase Tn5 is prevalently utilized in preparing various sequencing libraries due to its minimal sample input requirement and rapid processivity (Davies *et al.* 2000; Vaezeslami *et al.* 2007; Di *et al.* 2020; Lu *et al.* 2020). The Tn5 transposase dimer is distinctive for its unique tagmentation property: it can cleave double-stranded DNA (dsDNA) (D. R. Davies *et al.* 2000; Vaezeslami *et al.* 2007) and concurrently ligate specific adaptors to the resultant DNA ends, a process subsequently followed by PCR amplification with sequencing adaptors (Picelli *et al.* 2014; Hennig *et al.* 2018). This streamlined one-step tagmentation reaction has significantly simplified the experimental process, reducing both workflow duration and costs (Picelli *et al.* 2014; Hennig *et al.* 2018). Tn5 tagmentation has been widely adopted for detecting chromatin accessibility and interactions (Wu *et al.* 2016; Tan *et al.* 2018; Bentsen *et al.* 2020), as well as for enabling other types of sequencing experimentations (Gopalan *et al.* 2021).

In this study, we present a Tn5 transposase-assisted tagmentation technique for conducting WGS of a single *C. elegans* specimen. This efficient protocol requires a mere 20 min of hands-on time and costs <10 US dollars with a library size of 2 Gb. We show that the method is highly effective for pinpointing causal mutations in fertile mutants exhibiting defects in cilia or neurotransmitter secretion. Crucially, this method also facilitates the identification of mutations in mutants synthetically sterile with a variant analogous to the human BRAF (V600E) mutation. We demonstrate the applicability of this technique to one single-clone of yeast or algae. This method holds great potential for widespread use in WGS of chemically mutagenized model organisms and possibly mammalian cell lines. When combined with chemical mutagenesis, our approach offers a cost-effective and efficient means of generating and identifying missense variants, thereby enhancing the experimental exploration of missense variants across a range of species.

## Materials and methods

### Strains and genetics

*C. elegans* strains were maintained as described previously (Brenner 1974), on nematode growth medium (NGM) plates (3 g/L NaCl, 17 g/L agar, 2.5 g/L peptone, 1 mM CaCl_2_, 1 mM MgSO_4_, and 25 mM KPO_4_/pH 6) with OP50 feeder bacteria at 20°C. All the engineered *C. elegans* strains were genetic derivatives of the strain Bristol N2. Strains used in this study are summarized in Supplementary Table 1. Transformation of *C. elegans* to introduce the P*dyf-1::dyf-5* and P*unc-18::unc-18* rescue strains was performed by DNA injection as described (Mello *et al.* 1991), and the information of plasmids and primers are described in Supplementary Table 2. All animal experiments were performed following governmental and institutional guidelines. For OP50 culturation, one-clone of OP50 was streaked onto LB Agar plate (15 g/L agar, 10 g/L tryptone, 5 g/L yeast extract, 10 g/L NaCl) and incubated overnight at 37°C.

Motile *Chlamydomonas reinhardtii* wild-type strain 21gr was grown in standard Tris-acetate phosphate medium (2.42 g Tris, 1× Tris acetate phosphate (TAP) salt (0.375 g NH_4_Cl, 0.01 g MgSO_4_·7H_2_O, 0.05 g CaCl_2_·H_2_O), 0.114 g K_2_HPO_4_, 0.054 g KH_2_PO_4_, 0.1% Hutner's trace metals, 0.1% glacial acetic acid, 0.4 g NH_4_Cl, 0.05 g CaCl_2_·2H_2_O, and 0.1 g MgSO_4_·7H_2_O) in cycles of 12 h in fluorescent white light and 12 in darkness at 200 rpm at 20°C.

### Transposase-assisted tagmentation and library construction

For single-worm WGS, a solitary *C. elegans* specimen was employed as the experimental unit. In the case of single-clones of *Escherichia coli*, *Saccharomyces cerevisiae*, or *C. reinhardtii*, each microorganism was initially isolated and suspended in PBS buffer (137 mM NaCl, 2.7 mM KCl, 10 mM Na_2_HPO_4_, and 1.8 mM KH_2_PO_4_, Sigma, 806544). Subsequently, the isolated clones were enumerated using a cell counting board under a dissecting microscope, with ∼1,000 cells selected as representative samples within each experimental group. Samples were placed into separate PCR tubes containing 3 µl of lysis buffer (100 mM Tris–HCl pH 8.3, 500 mM KCl and, 15 mM MgCl_2_) with 0.3 mg/ml proteinase K (Thermofisher, 26160). Subsequently, freezing in liquid nitrogen for 1 min, followed by thawing in a 37°C water bath for 2 min, was performed for 3 times to lyse the cell protein. Afterward, the samples were subjected to a specific program in a PCR instrument, involving incubation at 65°C for 2 or 15 h, 95°C for 15 min, and a 4°C hold. An overnight incubation ensures a more complete release of DNA from chromatin. The resulting DNA samples were mixed with thawed reagents, including 1 µL 5× TruePrep Tagment Buffer L and 1.25 µL TruePrep Tagment Enzyme (Vazyme, TD502/TD503) Mix (Vazyme, TD502/TD503), and were then treated at 55°C for 10 min in a PCR instrument for DNA fragmentation. Following this, 1.25 µL of 5× Terminate Solution (Vazyme, TD502/TD503) was added, and the mixture was incubated at room temperature for 5 min. Subsequently, 2.5 µL of adaptors mix (Vazyme, TD202), 0.25 µL of TruePrep Amplify Enzyme (Vazyme, TD502/TD503), 2.5 µL of TruePrep Amplify Buffer (Vazyme, TD502/TD503), and 1 µL of double distilled water were added to the PCR tube on ice, followed by amplification through a PCR program with defined temperature cycles (Vazyme, TD502/TD503). Finally, amplified libraries each with individual adaptor pairs were purified using the PureLink Quick PCR Purification Kit (Invitrogen, #00995126).

### Forward genetic screens

We used forward genetic screens to isolate dye-filling defective animals, uncoordinated (Unc) animals, and multivulva animals. Adult animals were bleached by bleach buffer (1.26% NaHClO and 0.25 M NaOH) for 1.5 min to lyze worms and get eggs, which were then washed by M9 buffer (5.8 g/L Na_2_HPO4, 3.0 g/L KH_2_PO4, 0.5 g/L NaCl, and 1.0 g/L NH_4_Cl) for 3 times and hatched on NGM plates with fresh OP50. Worms were used as P0 and collected at the late L4 larval stage in 4 mL M9 buffer, and incubated with 180 mM EMS for 4 h at room temperature with constant rotation. Animals were then washed with M9 3 times and cultured under standard conditions. After 20 h, adult animals were bleached. Eggs (F1) were distributed and raised on ∼100 9-cm NGM plates, each containing 50–100 eggs. For the dye-filling defective animals, *osm-3::gfp(syd0199)* was used as P0. Adult F2 animals on each plate were collected and examined via the dye-filling assay (see Dye-filling assay below). For the Unc animals, *osm-3::gfp(syd0199)* was used as P0. F2 animals with Unc phenotypes were collected. For multivulva animals, the *lin-45 (syb4962)* animals were used as P0. F2 animals with multivulva phenotypes were collected. We identified mutations using WGS. We confirmed gene cloning using rescue experiments.

### Microinjection and transgenic strains

Transgenic or knockout *C. elegans* lines were generated by injecting the DNA constructs (Supplementary Table 3) into the gonads of the indicated worm strains. The co-injected selection marker was *pRF4(rol-6)*. At least 2 independent transgenic lines with a constant transmission rate (>50%) were propagated. Concentrations of DNA constructs used for generating rescue strains were 20 ng/µL.

### Dye-filling assay

The fluorescence dye DiI filling assay was widely used to assess the ciliary function and integrity. Animals that are dye-filling defective develop abnormal ciliary structures and are defective in animal behavioral assays, such as the osmotic avoidance assay and chemotaxis assay. Young adult worms were harvested into 100∼200 µL M9 buffer and mixed with equal volume dyes (DiI, 1,1′-dioctadecyl-3,3,3′,3′, -tetramethylindo-carbocyanine perchlorate, Sigma, 468495) at the working concentration (20 µg/mL), followed by incubation at room temperature in the dark for 30 min. Worms were then transferred to seeded NGM plates and examined for dye uptake 2 h later using a fluorescence stereoscope or fluorescence compound microscope. We observed at least 50 animals of each strain from 3 independent assays.

### RNAi by feeding

Young adult *C. elegans* hermaphrodites were anesthetized with 0.1 mM/L levamisole (Sigma, Y0000047) in M9 buffer, mounted on 3% agarose pads, and maintained at room temperature. Imaging was performed using a Zeiss Axio Observer Z1 microscope equipped.

### Imaging

Young adult *C. elegans* hermaphrodites were anesthetized with 0.1 mM/L levamisole (Sigma, Y0000047) in M9 buffer, mounted on 3% agarose pads, and maintained at room temperature. Imaging was performed using a Zeiss Axio Observer Z1 microscope equipped with an Andor iXon + EM-CCD camera, a Zeiss 10×/0.45 objective, and a Zeiss 100×/1.46 objective. Images were acquired by µManager (https://www.micro-manager.org). All the images were taken using identical settings. Image analysis and measurement were performed with ImageJ software (http://rsbweb.nih.gov/ij/).

### Next generation sequencing and data analysis

After library preparation, the DNA libraries were sent to the Novogen Corporation. The samples were subjected to an Illumina Nova seq for Paired-end 150 bp whole-genome sequencing (WGS) (Supplementary Table 4 for detailed strain and raw base count). Raw reads were assessed for quality with FastQC (version 0.11.9) and were trimmed using Trim_galore (version 0.4.4) to remove the adaptor sequence and low-quality reads. After that, clean reads were aligned to the reference genomes using BWA-MEM2 (version 2.2) with default parameters. PCR duplications were marked and removed with Picard (2.27.5 and OpenJDK 20.0.2) MarkDuplicates tool. Information about the depth and coverage of sequencing were generated by SAMtools and further analyzed by a Python script to generate the whole-genome scale coverage and depth plots. Reference genomes of every organism sequenced were listed in Supplementary Table 5, except *E. coli*, the sequence results of all the other organisms were compared to their up-to-date standard reference genomes. All the shell and python scripts used in the paper are available in the github repository: young55775/single-worm-sequencing (github.com).

## Results

### The single-worm WGS construction strategy

**Fig. 1.**
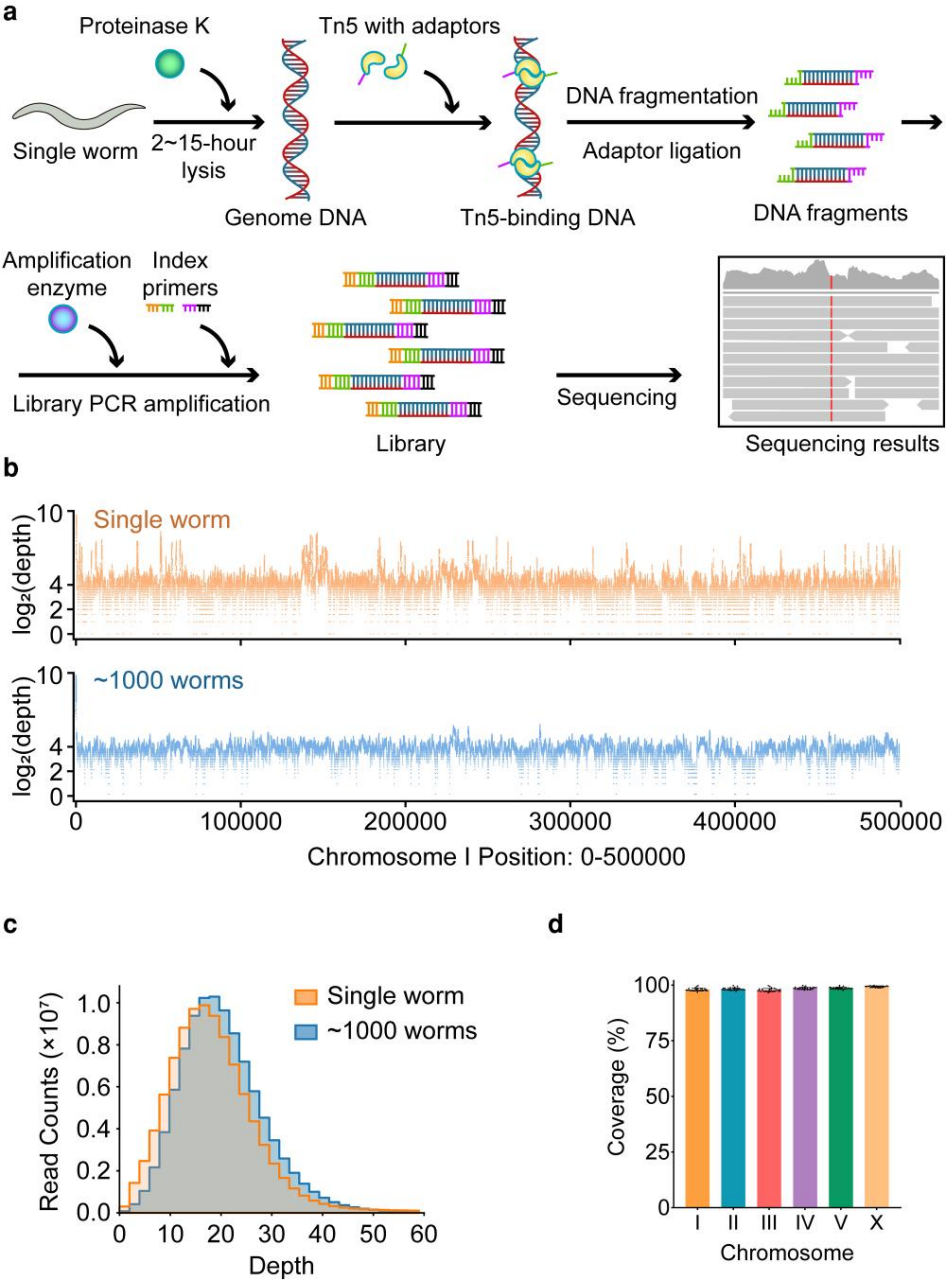
WGS by direct tagmentation of a single-worm. a) Scheme of single-worm WGS construction. b) The log_2_ (depth) distribution along chromosome I position 0–500,000 from a single-worm or about 1,000 worms. c) The read quantification of the depth from a single-worm or about 1,000 worms. d) Percentage of the coverage of each chromosome from a single-worm with 15-h PK digestion.

We introduce a rapid WGS library construction method utilizing a single-worm (Fig. 1a), comprising 3 primary components: *C. elegans* protein digestion, dsDNA tagmentation, and PCR amplification, culminating in an indexed library poised for sequencing. Initially, proteinase K is employed to digest a single-worm. While an extended digestion duration (e.g. 15 h) may facilitate comprehensive digestion of *C. elegans* proteins (Fig. 1b–d, Supplementary Fig. 1b), a concise 2-h digestion liberates sufficient DNA for subsequent experiments (Supplementary Fig. 1c and d). After the heat inactivation of proteinase K, the dsDNA undergoes tagmentation via the Tn5 transposase in the same tube, thereby appending partial sequencing adaptors to fragment ends. Thereafter, DNA polymerase amplifies the DNA fragments into a sequencing library. Amplified molecules were ∼150 bp longer than the tagmentation products, corresponding to the additional length from the adaptors added during index primer amplification (Supplementary Fig. 1a). This illustrates that Tn5 tagmentation of the *C. elegans* genomic DNA provides a feasible strategy for preparing a WGS library from an individual worm. The entire workflow requires just a single test tube and ∼5 h, with a hands-on time of below 30 min.

### The single-worm WGS results

In our analysis of the WGS data, we discovered that the sequencing results encompassed roughly 98% of the genome (Fig. 1d), with an average depth of 20× (Fig. 1b and c), a coverage that is comparable to existing WGS results obtained using ∼1,000 worms (Supplementary Fig. 2b for comparison). Extending our initial success to an additional 29 individual worms, we achieved highly reproducible WGS results (Supplementary Fig. 1b). Furthermore, when we plotted the WGS coverage data for all 29 worms on each chromosome, we observed consistently high, unbiased coverage, further attesting to the robustness of the method (Fig. 1d).

### The single-worm WGS identified mutations responsible for mutant phenotypes

**Fig. 2.**
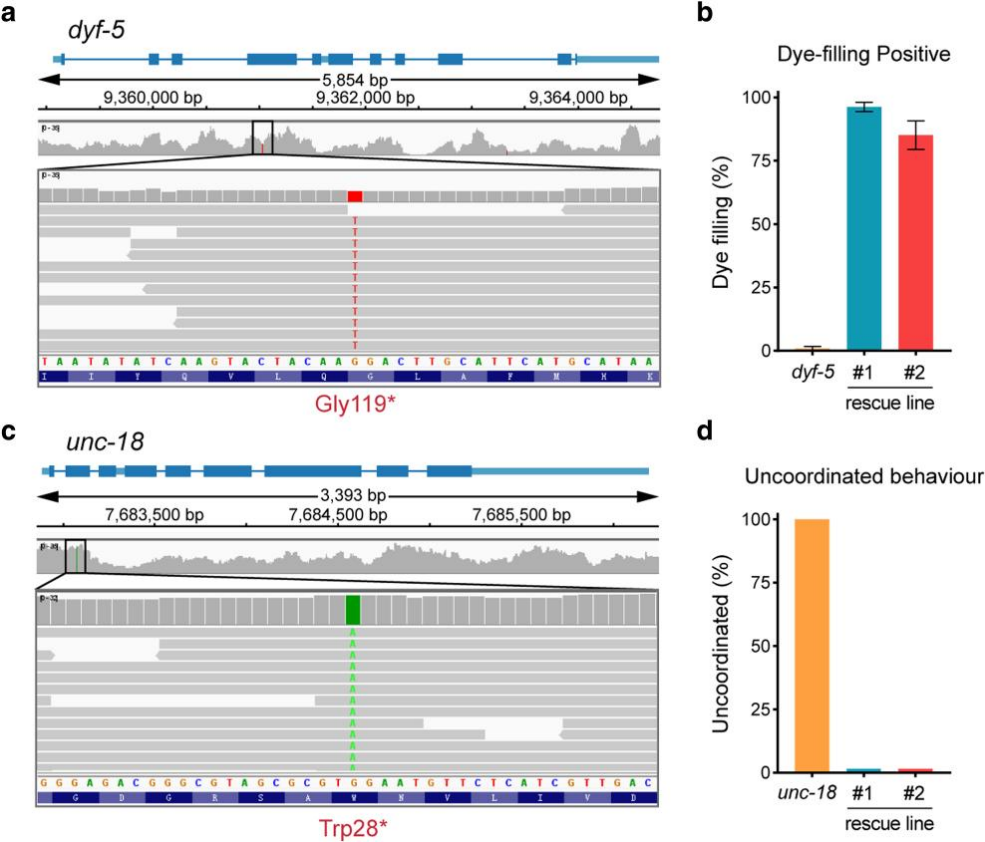
The single-worm WGS identified mutations responsible for mutant phenotypes. a) An integrative genomics viewer (IGV) window shows the mutation site of the *dyf-5* mutant. b) Percentages of Dye-filling positive *dyf-5* mutant and the *dyf-5* rescue worms. *n* > 50. c) An IGV window shows the mutation site of the *unc-18* mutant. d) Percentages of Unc behavior *unc-18* mutant and the *unc-18* rescue worms. *n* > 50.

We explored whether the WGS results obtained from a single-worm would enable the identification of genetic mutations induced by EMS mutagenesis. *C. elegans* utilize their sensory cilia to engage with environmental stimuli (Hedgecock, Culotti, Thomson, and Perkins 1985; Ou *et al.* 2005). In wild-type worms, which develop normal cilia, a carbocyanine dye, DiI, can be absorbed through sensory cilia (Hedgecock *et al.* 1985). Conversely, cilia mutants, which fail to fill with DiI, exhibit a dye-filling defect (Dyf) phenotype, making the dye-filling assay a powerful tool for isolating ciliary mutants (Hedgecock *et al.* 1985). We found that the *cas2885* strain did not uptake DiI, and our WGS results from a single mutant animal revealed that *cas2885* harbors a newly acquired stop codon in *dyf-5* (Fig. 2a), which encodes a ciliary kinase essential for ciliary length regulation (Burghoorn *et al.* 2007; Omori *et al.* 2010). To validate that this mutation is responsible for the Dyf phenotype, we injected the wild-type (WT) *dyf-5* cDNA under the control of the ciliated neuron-specific promoter, P*dyf-1*, into the *cas2885* strain. We demonstrated successful restoration of the Dyf defects in 2 independent transgenic lines (Fig. 2b).

Similarly, we employed EMS mutagenesis to generate a cohort of animals exhibiting Unc movement. Among them, our single-worm WGS identified a point mutation leading to an ectopic stop codon in the *unc-18* gene (Fig. 2c), which regulates neurotransmitter secretion (Sassa *et al.* 1999). By introducing the wild-type *unc-18* gene into the *cas4401* strain carrying this mutation, we observed a complete rescue of its Unc phenotype in 2 independent transgenic lines (Fig. 2d). Thus, the examples of *dyf-5* and *unc-18* validate that the single-worm WGS results enable us to identify genetic variants responsible for animal phenotypes.

### The single-worm WGS identified mutations from a sterile mutant

**Fig. 3.**
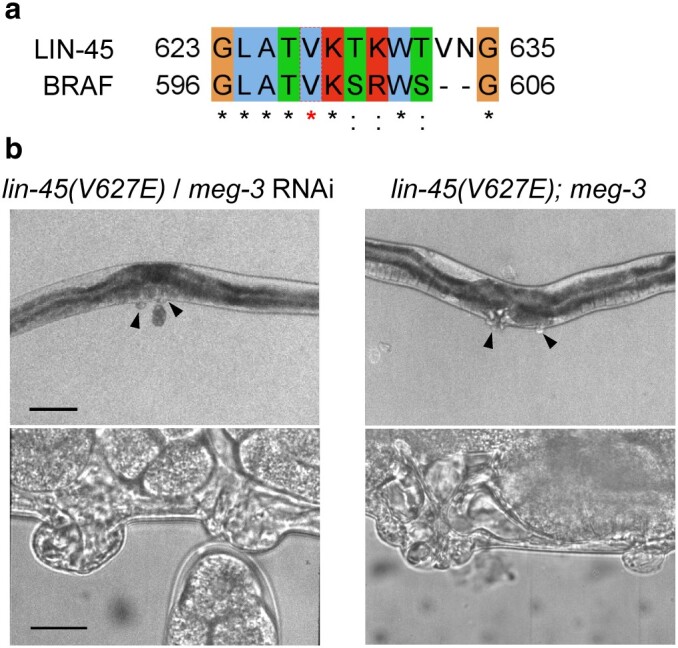
The single-worm WGS identified mutations from a sterile mutant. a) An Jalview window shows a part of the alignment between the human BRAF and *C. elegans*  LIN-45. b) Representative images of the vulva of *lin-45(V627E)* treated with *meg-3* RNAi and *lin-45(V627E)*; *meg-3*. For 10× magnification images, scale bar is 100 µm. For 100× magnification images, scale bar is 10 µm.

We investigated the potential of our method in single worms that are progeny-deficient. Specifically, we performed a genetic enhancer screen on a strain with a LIN-45 missense mutation V627E, analogous to the pathogenic BRAF (V600E) mutation in humans (Fig. 3a). The *BRAF* gene, a critical component of the Rat Sarcoma/Rapidly Accelerated Fibrosarcoma/Mitogen-Activated Protein Kinase Kinase/Extracellular Signal-Regulated Kinase (RAS/RAF/MEK/ERK) signaling pathway, is pivotal in regulating cell division and differentiation (H. Davies *et al.* 2002; Shen *et al.* 2013; Lavoie *et al.* 2018). LIN-45 serves as the *C. elegans* orthologue of BRAF (Kim, Underwood, Greenwald, and Shaye 2018). The V600E mutation in BRAF leads to excessive activation of the kinase, triggering uncontrolled cell division and contributing to cancer development (H. Davies *et al.* 2002; Li *et al.* 2006; D. Liu, Liu, Condouris, and Xing 2007; Sanchez-Torres, Viteri, Molina, and Rosell 2013; Ritterhouse and Barletta 2015). Employing genome editing, we created a *C. elegans* strain with the corresponding *lin-45(V627E)* mutation. This mutation manifested as a protruding vulva phenotype with ectopic cells due to overproliferation.

During our enhancer screen across ∼20,000 haploid genomes, we isolated 30 suppressors from the F2 generation displaying a multivulva phenotype, likely due to additional unregulated cell divisions. Remarkably, only 9 of these suppressors produced viable progeny exhibiting an intensified vulva phenotype; the rest were sterile. We conducted single-worm whole-genome sequencing (WGS) on these sterile lines and discovered a stop-gain mutation in the *meg-3* gene, a known regulator of cell fate (Lee *et al.* 2020). When *meg-3* was knocked down via RNAi in the LIN-45 V627E strain, about 4.8% of the 96 progenies showed the multivulva phenotype (Fig. 3b). While the penetrance was low, this phenotype was absent in *meg-3* RNAi-treated wild-type animals and in the negative control RNAi *lin-45(V627E)* strain. To further examine this, we used CRISPR–Cas9 to generate *meg-3* knockout strains in the *lin-45(V627E)* background. We found that *meg-3* null mutants with the *lin-45(V627E)* strain developed an enhanced vulva phenotype (Fig. 3b) but were unable to produce offspring. These results demonstrate how single-worm WGS can effectively pinpoint causative mutations in sterile strains.

### Effective single-clone WGS in bacteria, yeast, and algae

**Fig. 4.**
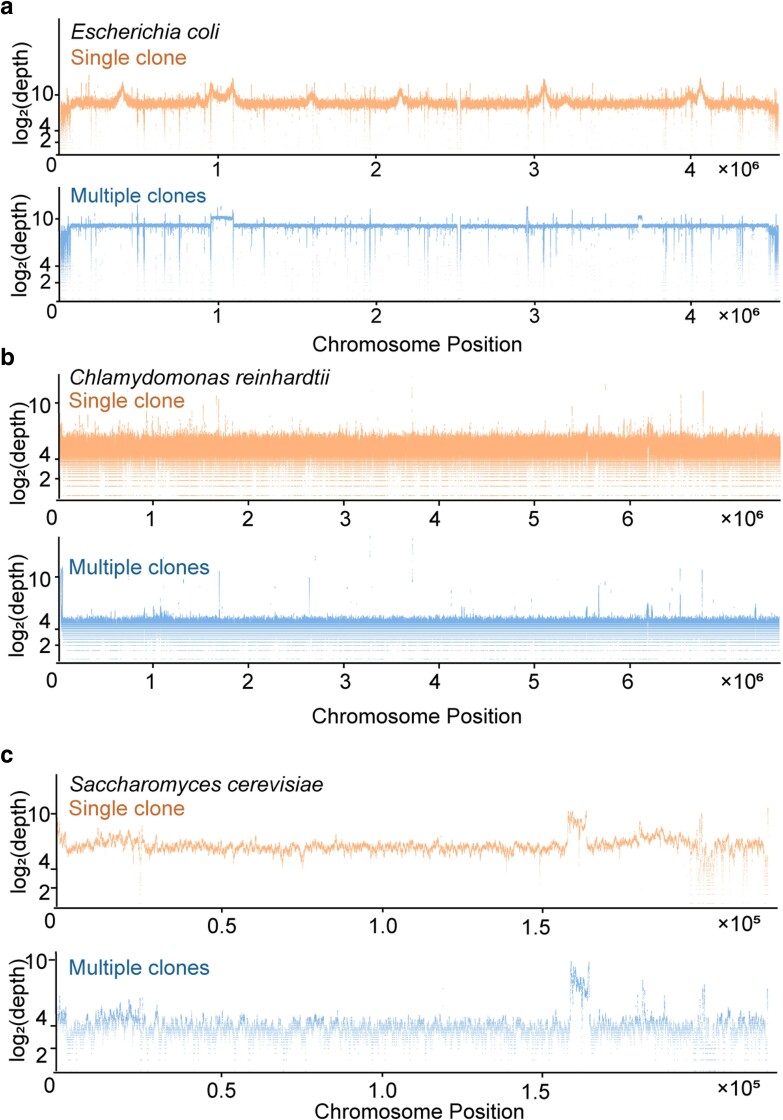
Effective single-clone WGS in bacteria, yeast, and algae. a) The log_2_(depth) distribution along the chromosome from a single-clone or multiple-clones of *E. coli*. b) The log_2_(depth) distribution along Chromosome I from a single-clone or multiple-clones of *C. reinhardtii*. c) The log_2_(depth) distribution along Chromosome I from a single-clone or multiple-clones of *S. cerevisiae*.

To evaluate the applicability of our method in other species, we wondered about its efficacy in performing WGS on single-clones of *E. coli*, *S. cervisiae* and *C. reinhardtii*, each comprising ∼1,000 cells—comparable to the 959 somatic cells of an individual *C. elegans*. The identical protocol adeptly generated the WGS library using the cell number equivalent to ones from a single-clone, the sequencing of which yielded a coverage rate and depth analogous to that of a single *C. elegans* (Fig. 4a–c, Supplementary Figs. 3 and 4).

## Discussion

In summary, our findings demonstrate that Tn5 transposase-assisted tagmentation can facilitate the development of a methodology capable of generating DNA libraries for WGS from a single-worm. The same method is applicable across other species, such as a single-clone of yeast or algae. The implementation of the protocol is notably straightforward. The single-worm PCR protocol, frequently utilized for *C. elegans* genotyping, is often considered an entry-level experiment for novice researchers in *C. elegans* research groups or even in educational laboratories (Barstead, Kleiman, and Waterston 1991; Williams, Schrank, Huynh, Shownkeen, and Waterston 1992). Our single-worm WGS protocol merely introduces an additional Tn5 reaction step in the same tube, underscoring its operational simplicity. As outlined in the Materials and Methods section, the single-worm WGS library construction is accomplished within a 15-µL reaction volume, with reagent costs amounting to only $3. Given that the *C. elegans* genome is ∼100 Mb in size, generating 2 Gb of data for a 20× coverage rate comes at a minimal cost of $7. Notably, many labs opt to sequence the genome at a 5× coverage rate, effectively halving the sequencing cost with 1 Gb of data, reducing the total to $10 or even less. Considering that all reactions can be conducted in a tube within a 96-well plate, after a single-worm or clone is placed into the tube, all subsequent steps can potentially be automated. This not only minimizes experimental errors but also enhances scalability. Given that Tn5 transposases also act on DNA/RNA hybrids, protocols for constructing an RNA-seq library have been established in previous studies (Di *et al.* 2020; Lu *et al.* 2020). Consequently, we posit that generating both WGS and RNA-seq libraries from a single-worm is plausible, thereby potentially furnishing both genomic and transcriptomic information from an individual *C. elegans*.

While the per-missense variant generation in *C. elegans* is estimated to be mere cents, it is imperative to note that this production strategy leans on random mutagenesis, not on a precision-targeted knock-in substitution. The probability of obtaining a desired missense variant is tethered to the size of the worm library fortified with WGS information. On average, *C. elegans* proteins encompass about 470 amino acids, and its genome harbors ∼20,000 protein-encoding genes (Yates *et al.* 2020), this culminates in around 9.4 million residues within the *C. elegans* proteome. Given that each chemically mutagenized worm typically carries around 91 missense mutations (Thompson *et al.* 2013), conducting WGS on 0.1 million *C. elegans* mutant strains could, in probability, mutate each residue once. This results in single-fold mutation coverage at an estimated expenditure of 1 million US dollars. Although EMS mutagenesis typically favors GC to AT conversions (Greene *et al.* 2003), the incorporation of an additional mutagen, N-ethyl-N-nitrosourea (ENU) (Russell *et al.* 1979), is frequently employed to formulate “cocktail” mutagens (Thompson *et al.* 2013), thereby augmenting the diversity of mutation forms. Hence, with the allocation of additional resources, achieving comprehensive coverage of various types of missense variants becomes plausible. While this protocol is established in simpler model organisms, we anticipate its broad application across numerous human cell lines, including those with a haploid genome that are primed for chemical mutagenesis-based forward genetic screens.

The single-worm/-clone WGS methodology stands poised to expedite the functional study of missense variants identified within the human proteome, thereby harboring the potential to advance precision genomic medicine to a nucleotide-resolution tier. Alterations to individual amino acid residues within a protein may lead to distinct dysfunctions at biochemical, cellular, and organismal levels, each likely demanding unique interventional approach. This predicament underscores an emerging field, termed “functional residuomics,” which endeavors to provide residue-resolution functional insights into the proteomic landscape.

The generation of missense variants in model organisms constitutes a foundational step in functional residuomic studies. Organisms harboring missense variants swiftly provide empirical evidence, crucial for differentiating benign from pathogenic variants. Characterizing the cellular and organismal impacts of pathogenic variants enables the acquisition of invaluable mechanistic insights into the interplay between genetic anomalies and symptomatic manifestations. Crucially, organisms that carry pathogenic variants present a starting point for executing genetic suppressor screens. This can illuminate strategies for phenotype rescue, potentially paving the way for effective clinical interventions and informing drug discovery endeavors.

If millions of strains are sequenced via WGS, a paramount challenge emerges in the storage and distribution of the sequenced strains. Presently, such reagents are deposited into genetic centers, like the *C. elegans* Genetic Center, which has been distributing strains for over four decades. Nevertheless, nonprofit resource centers, bounded by limited government support, cannot expand indefinitely, a constraint equally applicable to commercial services like Addgene that distributes published plasmids. No institute akin to these centers possesses the capacity to store and distribute millions of strains with distinct genetic backgrounds, especially as these numbers perpetually augment. A decentralization mechanism, reminiscent of eBay, may be a forward-thinking solution to navigate this predicament: each laboratory conducts their WGS and data analysis, depositing the sequencing information into a public platform or database. While a lab might concentrate on a missense mutation of interest to their work, others might scour the database for additional valuable variants. This platform not only supports the exchange of WGS information but also fosters the trade of strains, allowing laboratories to negotiate expenses, thereby encouraging a collaborative and mutually beneficial scientific environment.

## Supplementary Material

jkae094_Supplementary_Data

## Data Availability

Strains and plasmids are available upon request. The authors affirm that all data necessary for confirming the conclusions of the article are present within the article, figures, and tables. Sequencing data were analyzed by FastQC (https://github.com/s-andrews/FastQC), Trim_galore (https://github.com/FelixKrueger/TrimGalore), BWA-MEM2 (https://github.com/bwa-mem2/bwa-mem2), Picard (https://github.com/broadinstitute/picard), and SAMtools (https://github.com/samtools/samtools). Sequencing data were published on a public repository (DOI for *C. elegans* sequencing data: 10.5281/zenodo.11141199; DOI for *E. coli*, *S. cervisiae*, and *C. reinhardtii* sequencing data: 10.5281/zenodo.11141386). All the shell and python scripts used in the paper are available in the github repository (https://github.com/young55775/single-worm-sequencing). Supplemental material available at G3 online.
