# Supplementary material for: Transposase-assisted tagmentation: an economical and scalable strategy for single-worm whole-genome sequencing
Source: G3 (Bethesda). 2024 Jun 10;14(7):jkae094. doi: 10.1093/g3journal/jkae094 (PMC11228870; doi:10.1093/g3journal/jkae094)
Supplement: jkae094_Supplementary_Data [file jkae094_supplementary_data.docx]

**
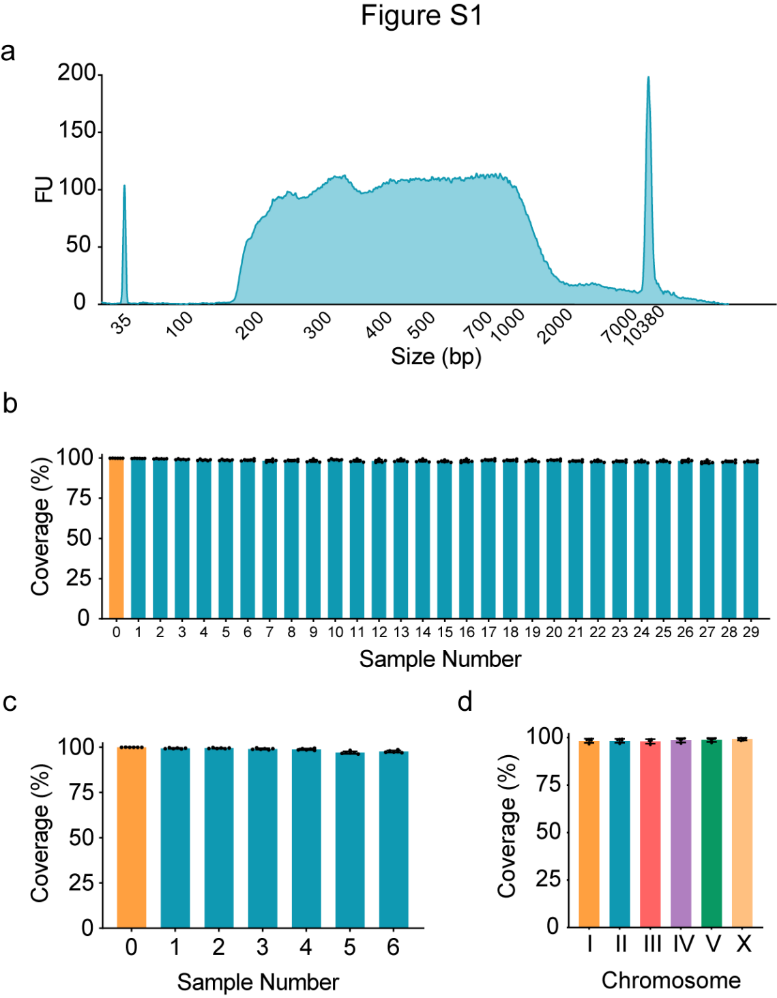
**

**Figure S1. Quality of single-worm WGS.**

a. Representative Bioanalyzer electropherogram of a single-worm whole-genome library. b-c. Percentage of the coverage of each chromosome from individual worm sequencing data. b. The orange bar represents one traditional WGS data, blue bars represent 29 single-worm WGS data from worms with 15-hour PK digestion. c. The orange bar represents one traditional WGS data, blue bars represent 29 single-worm WGS data from worms with 2-hour PK digestion. d. Percentage of the coverage of each chromosome from a single worm with 2-hour PK digestion.

**
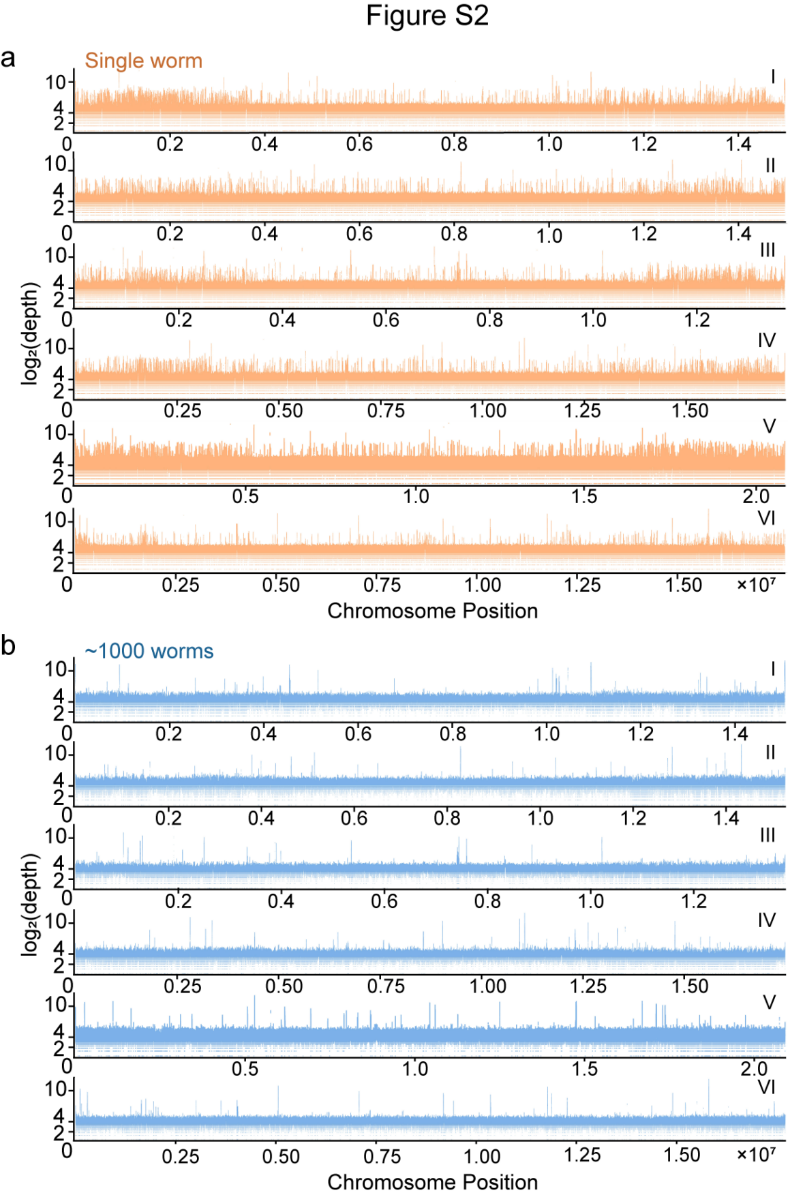
**

**Figure S2. Quality comparison between single-worm WGS and traditional WGS.**

a. The log_2_(depth) distribution along individual chromosomes from a single *Caenorhabditis elegans*. b. The log_2_(depth) distribution along individual chromosomes from about 1000 *Caenorhabditis elegans*.


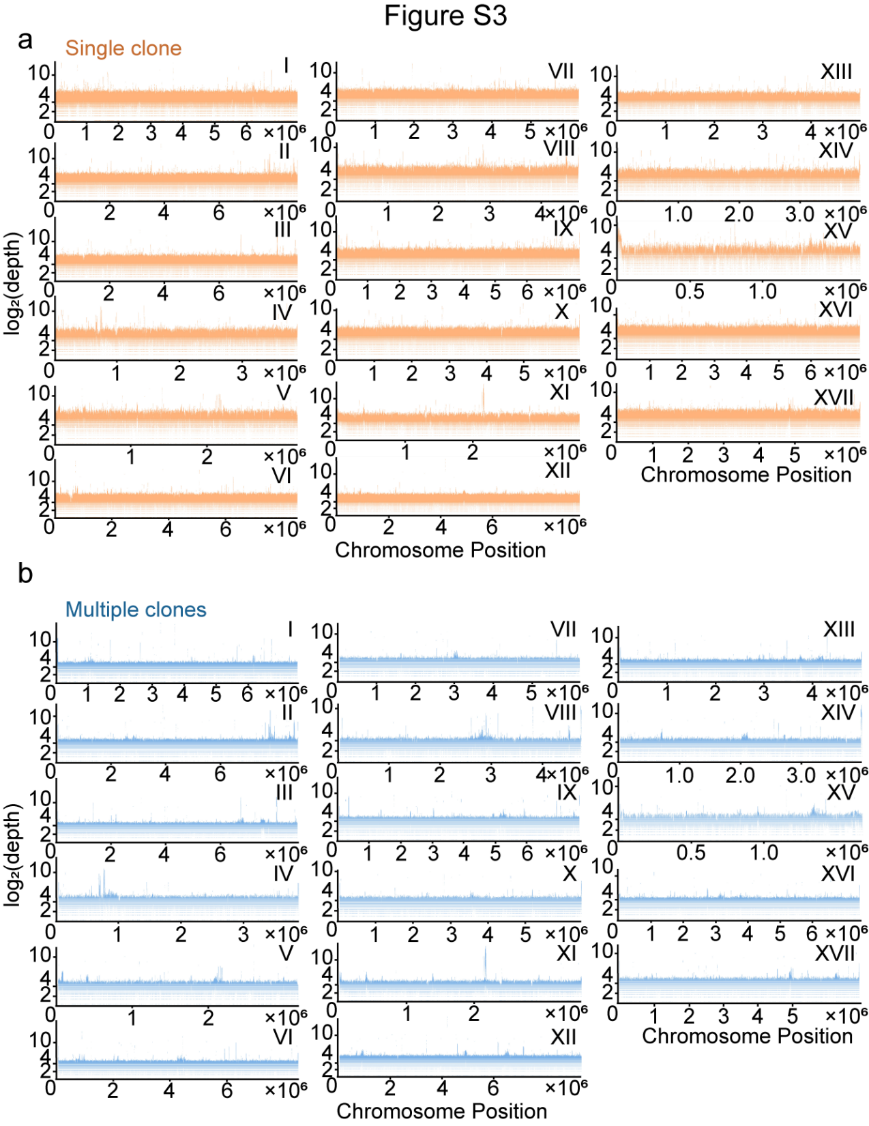


**Figure S3. Quality comparison between single-clone WGS and traditional WGS in** ***Chlamydomonas reinhardtii*.**

a. The log_2_(depth) distribution along individual chromosomes from a single clone of *Chlamydomonas reinhardtii*. b. The log_2_(depth) distribution along individual chromosomes from multiple *Chlamydomonas reinhardtii*.


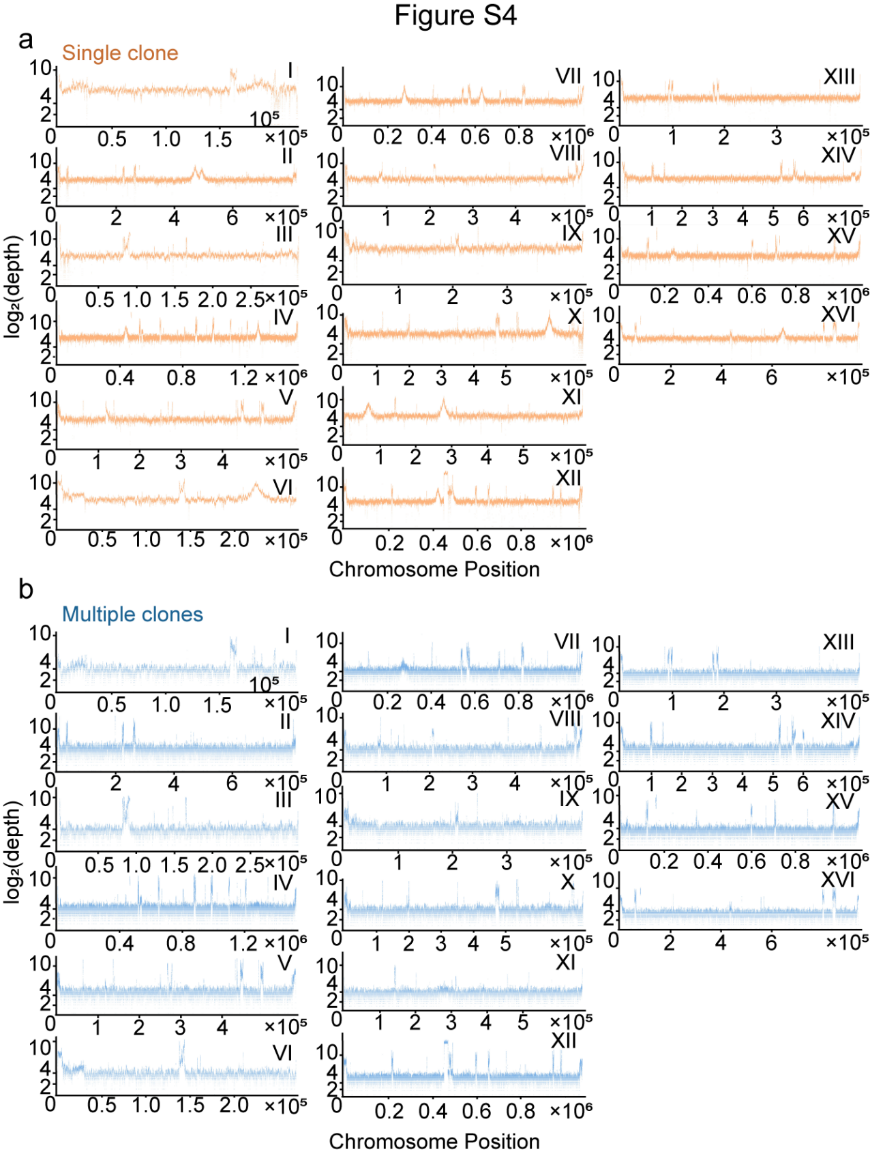


**Figure S4. Quality comparison between single-clone WGS and traditional WGS in** ***Saccharomyces cerevisiae*.**

a. The log_2_(depth) distribution along individual chromosomes from a single clone of *Saccharomyces cerevisiae*. b. The log_2_(depth) distribution along individual chromosomes from multiple *Saccharomyces cerevisiae*.**Table S1. *C. elegans* Strains in this study.**

| **Strain Name** | **Genotype** | **Method** |
| --- | --- | --- |
| SYD0199 | *osm-3(syd0199[osm-3::gfp KI])* |  |
| *cas2885* | *dyf-5(G119*); osm-3(syd0199[osm-3::gfp KI])* | EMS screen |
| *Cas4401* | *unc-18(W28*); osm-3(syd0199[osm-3::gfp KI])* | EMS screen |
| *cas2940* | *dyf-5(G119*); osm-3(syd0199); Ex[dyf-1p::dyf-5 + rol-6(su1006)]* line 1 | Microinjection |
| *cas2941* | *dyf-5(G119*); osm-3(syd0199); Ex[dyf-1p::dyf-5 + rol-6(su1006)]* line 2 | Microinjection |
| *cas2942* | *unc-18(W28*); osm-3(syd0199); Ex[unc-18p:: unc-18 + rol-6(su1006)]* line 1 | Microinjection |
| *cas2943* | *unc-18(W28*); osm-3(syd0199); Ex[unc-18p:: unc-18 + rol-6(su1006)]* line 2 | Microinjection |

**Table S2. Plasmids, PCR fragments, and Primers in this study.**

| **Plasmid/PCR fragment** | **Forward Primer** | **Reverse Primer** | **Notes** |
| --- | --- | --- | --- |
| pDONR-P*dyf-*  *1::dyf-5b*gDNA | ATGTCATCGGCTGTTAAACTTGCT | CTATTTTACATATCTGAAAGCATTTC | *dyf-5b* genomic fragment was amplified from N2 and inserted into pDONR plasmid via In-Fusion Advantage PCR Cloning Kit. |
| *unc-18p::unc-18* | CGCCCGAGCTCCAATCTATCC | ACTGCAAAGCTAATGTGGAGTTGAATG | The *unc-18p::unc-18* was amplified from N2 genomic DNA |

**Table S3. CRISPR-Cas9 Targets in this study.**

| **Gene** | **CRISPR-Cas9 targets (PAM)** | **Application Description** |
| --- | --- | --- |
| *meg-3* | GATTGCTACGACTACTTGAG | *meg-3* knock-out |
|  | CTCAAGTAGTCGTAGCAATC | *meg-3* knock-out |

**Table S4. Species for sequencing**

| **Taxonomy** | **strain** | **Reference genome** |
| --- | --- | --- |
| *Caenorhabditis elegans* | N2 | https://ftp.ensembl.org/pub/release-110/fasta/caenorhabditis_elegans/dna/Caenorhabditis_elegans.WBcel235.dna.toplevel.fa.gz |
| *Saccharomyces cerevisiae* | NYM51 | https://ftp.ensembl.org/pub/release-110/fasta/saccharomyces_cerevisiae/dna/Saccharomyces_cerevisiae.R64-1-1.dna.toplevel.fa.gz |
| *Escherichia coli* | OP50 | https://www.ncbi.nlm.nih.gov/datasets/genome/  GCF_013456775.1/ |
| *Chlamydomonas reinhardtii* | 21gr | https://ftp.ensemblgenomes.ebi.ac.uk/pub/plants/release-57/fasta/chlamydomonas_reinhardtii/dna/Chlamydomonas_reinhardtii.Chlamydomonas_reinhardtii_v5.5.dna.toplevel.fa.gz |

**Table S5. Softwares for sequencing analysis**

| **Software** | **Version** | **Link** |
| --- | --- | --- |
| FastQC | 0.11.9 | https://github.com/s-andrews/FastQC |
| Trim_galore | 0.4.4 | https://github.com/FelixKrueger/TrimGalore |
| BWA-MEM2 | 2.2 | https://github.com/bwa-mem2/bwa-mem2 |
| Picard | 2.27.5 | https://github.com/broadinstitute/picard |
| Samtools | 1.18 | https://github.com/samtools/samtools |
